# The young osteoarthritic knee: dilemmas in management

**DOI:** 10.1186/1741-7015-11-14

**Published:** 2013-01-18

**Authors:** Paul M Sutton, Edward S Holloway

**Affiliations:** 1 Department of Trauma and Orthopaedics, Northern General Hospital, Sheffield Teaching Hospitals NHS Trust, Herries Road, Sheffield, S5 7AU, UK

**Keywords:** high tibial osteotomy, osteoarthritis, total knee replacement, unicondylar knee replacement.

## Abstract

As a result of increasing life expectancies, continuing physical careers, lifestyles into later life and rising obesity levels, the number of younger patients presenting with osteoarthritis (OA) of the knee is increasing. When conservative management options have been exhausted, the challenge for the orthopedic surgeon is to offer a procedure that will relieve symptoms and allow a return to a high level of function but not compromise future surgery that may be required as disease progresses or prostheses fail and require revision. We discuss the options available to this group of patients and the relative benefits and potential negative points of each. Total knee replacement (TKR) in the young patient is associated with high risk of early failure and the need for future revision surgery. After TKR, most surgeons advise limitation of sporting activities. If osteoarthritis is limited to only one compartment in the knee there may be surgical options other than TKR. Osteotomy above or below the knee may be considered and works by redirecting the load passing through the joint into the relatively unaffected compartment. A unicompartmental knee replacement (UKR) or patella-femoral joint (PFJ) replacement only replaces the articular surfaces in the affected compartment, leaving the unaffected compartments untouched with better preservation of the soft tissues. Which of these options is best for a particular patient depends upon the patient's symptoms, precise pathology, lifestyle, and expectations.

## Background

The number of young patients seeking medical consultation for symptoms relating to osteoarthritis (OA) of the knee is increasing [1]. This is thought to be due to a combination of factors [2]. Longer life expectancy also means that the proportion of the population continuing physically demanding careers and sporting lifestyles into their fifth, sixth, and even seventh decades is increasing [3]. In addition to these risks, there are rising levels of obesity and there is clear evidence that the risk of OA is increased with obesity [4]. Allied to the increasing rates of OA are patient expectations that a return to previous levels of activity should be possible following injury or trauma.

Osteoarthritis is a dynamic and metabolically active disease that involves all tissue components of the joint: bone, cartilage, synovium, muscle, and ligament. The key pathological features are of articular cartilage softening, fibrillation, and then ulceration leading to sclerosis and eburnation of subchondral bone. Articular cartilage degradation leads to release of inflammatory mediators and further joint damage due to this inflammatory component, thus the frequent reference to 'degenerative arthritis' is incorrect. Subchondral cyst and osteophyte formation are later features [5].

Patients with OA of the knee present with symptoms that include joint pain, swelling, stiffness, crepitus, loss of function, and reduced quality of life [6]. The initial management of OA of the knee should be non-operative, however, these measures often provide only limited and temporary benefit. Not until these options have been exhausted should surgical options be explored. Non-operative strategies may include patient education, exercise, weight loss, bracing, analgesia, non-steroidal anti-inflammatory drugs (NSAIDs) and possibly intra-articular (IA) injections. Although many of these treatment methods are employed the evidence for their benefit is mixed.

Meta-analyses have shown the benefit of muscle strengthening and aerobic exercise in the management of OA [7,8] but have highlighted the importance of patient compliance. The medical benefits of weight loss for the obese patient are clear. While it seems logical that weight loss should also relieve the pain associated with OA the evidence that weight loss can delay or even reverse symptoms associated with OA of the knee remains mixed [9]. There are studies that have shown bracing to be effective [10]. Bracing aims to reduce the biomechanical load in the affected compartment of the knee, or improve symptoms by reducing perceived instability. Although potentially effective, bracing is poorly tolerated by patients due to discomfort or skin complications, such as blistering, and there is evidence that compliance with long-term bracing for orthopedic conditions is poor.

Analgesic regimes have been proven to effectively improve the pain associated with OA. Paracetamol is effective in reducing the pain associated with OA of the knee [11] but less so than NSAIDs. Systemic non-steroidal anti-inflammatory drugs are however associated with an increased risk of side effects such as gastrointestinal disturbance.

Intra-articular corticosteroids are widely used to manage the symptoms of osteoarthritic joints. Several studies and a Cochrane Review have shown the treatment to be beneficial versus placebo in the knee, but that there is little evidence to show that this benefit lasts beyond 4 weeks [12,13]. The 2009 Cochrane review [14] analyzed the evidence for hyaluronan (HA) and hylan derivatives for viscosupplementation of the knee. The authors concluded that these products were comparable in efficacy to systemic forms of active intervention but with more local reactions and fewer systemic adverse events. The HA products have a more prolonged effect than IA corticosteroids. The numerous different HA preparations and administration regimes make a comparison of cost with corticosteroid difficult, but the raw cost of the HA is significantly more expensive than corticosteroid. Infection following joint injection is a rare but recognized complication and there is evidence of a higher risk of joint infection following TKR in patients who have had IA corticosteroid treatment. This makes some surgeons wary of offering IA injections to patients who may be a candidate for future joint replacement surgery.

Older patients with OA of the knee are successfully managed with TKR. These have been implanted in one form or another for over 50 years [15]. Over time both prostheses and surgical techniques have evolved [16] such that 15-year survival rates of up to 98% have been reported in older populations [17]. For young people who may have a long life expectancy the concern is that the longevity of the prosthesis does not match that of the patient and complex revision surgery will be inevitable. The longer life expectancy of younger patients may be compounded by higher demand resulting in lower implant survival rates. Julin *et al. *[18] reported on the survival rates of TKR between different age groups from the Finnish Arthroplasty Register. Follow-up of 32,019 patients showed that 5-year survival rates were 92% and 95% in patients aged ≤ 55 and 56 to 65 years, respectively, compared to 97% in patients who were > 65 years of age (*P *< 0.001). These differences remained statistically different once differences in implant and fixation type, sex, and diagnosis were adjusted for. W-Dahl *et al. *[19] similarly interrogated the Swedish Register and found a 10-year cumulative revision rate for patients younger than 55 years of age of 9%. Odland *et al. *[20] report 10-year outcomes from a cohort of 59 active patients (67 knees) of 55 years or younger. A total of 65% of patients were still performing moderate labor or sport activities but 16% had undergone revision for wear and/or osteolysis.

In addition to the issues of implant survival and complexity of future revision surgery, function after TKR may be a concern for younger and more active patients. Most surgeons advise against high-impact activities following TKR, which precludes patients from participating in a wide range of sports. The list of activities 'not recommended' by surgeons after TKR according to Knee Society surveys in 1999 and 2005 is decreasing [21] and there is a paucity of evidence to guide patient advice about sporting activities after this procedure. There are small-scale series published of patients returning to high-level tennis [22] and golf [23] but these are in very select patient groups. The principle behind the advice given to patients to avoid high-impact activities is that implant wear is related to joint use not duration of implantation [24].

Regardless of etiology the aims of surgical treatment for early OA remain the same: to provide pain relief and enable a return to a high level of function. Additionally, when considering surgical treatment options the ability to perform further surgery if disease progresses or implants fail should be taken into account.

We will discuss the surgical options available to treat OA of the knee in young adults. Defining 'young' in this context is not simple and will clearly depend on both the patient's chronological and biological age, however, for the purposes of this discussion we consider patients less than 55 years of age.

Historically, other than TKR the main surgical options for these patients were considered to be osteotomy or unicompartmental tibio-femoral knee replacement (UKR). However, advances in arthroscopic techniques and biologic treatments have opened up other potential avenues, as have patella-femoral joint (PFJ) replacements. We will discuss these treatments separately and consider the relative benefits and potential risks of each.

## Discussion

### Osteotomy

An osteotomy is a surgical procedure to cut a bone. The cut bone from the osteotomy is then removed or fixed into a different position. In the normal knee, loads that are over twice body weight are imposed on the tibio-femoral joint during walking. This rises to over five times body weight in activities such as descending stairs. In patients with neutral alignment, on standing the mechanical axis of the lower limbs passes just medial to the anatomical center of the knees and during single-leg stance up to 75% of the knee joint's load passes through the medial tibial plateau [25]. This load is increased further in patients with varus alignment (bow leggedness). The most common pattern of symptomatic OA within the knee is articular cartilage degeneration predominantly in the medial compartment. This results in a varus deformity further increasing load transmission through the already degenerate compartment. A high tibial osteotomy (HTO) aims to redirect the mechanical axis in order to 'offload' the medial compartment and transfer more of the load through the relatively preserved articular cartilage in the lateral compartment. This has been shown to reduce pain and slow disease progression [26]. Coventry [27] in the 1960s recommended a postoperative correction to 10° to 13° of valgus. Fujisawa *et al*. describe the degree of correction differently and have suggested that best results from HTO are obtained when the mechanical axis line passes through a point 30% to 40% across the lateral tibial plateau, with the inner edge marking 0% and the outer edge 100% of the plateau [28].

Osteotomies around the knee have been performed since the beginning of the 20th century. There are many different techniques for performing the osteotomy, augmenting, and then fixing the bone. The osteotomy may be wedge or dome shaped, and if a wedge, either an opening or closing wedge. In our experience a dome-shaped osteotomy is rarely performed today and is largely of historic interest. In the 1970s, Coventry and Insall popularized an HTO procedure similar to that widely performed today [27,29,30].

Under radiographic control, a closing-wedge HTO makes two bone cuts such that a wedge of bone is taken from the lateral side of the proximal tibia. The potential drawbacks to this technique are that it involves dissection of the tibialis anterior, it may necessitate an osteotomy of the fibula or disruption of the proximal tibio-fibula joint both of which are associated with a risk of damage to the common peroneal nerve. This technique also requires careful preoperative planning to ensure a proper correction. Some surgeons prefer a medial sided approach and an opening-wedge osteotomy. With this technique the osteotomy may be gradually opened until the desired correction is achieved and the resulting wedge is held open the precise amount, typically with a plate and screws. The wedge-shaped defect may be left to fill naturally, bone grafted with the patient's own bone, or filled with a synthetic bone graft substitute, such as tricalcium phosphate. There are no significant differences in the outcomes of a closing versus opening wedge osteotomy in comparative studies [31,32]. Recent developments allow the use of 'computer navigation' to accurately perform the desired correction, however, to date there is no evidence that this technique is associated with an improved outcome compared with traditional techniques for determining the correction. The cancellous bone in the proximal tibia heals quickly and patients are often able to weight bear immediately after surgery. We are aware that some surgeons routinely partially weight bear patients for 6 weeks following HTO, allowing full-weight bearing when radiographic evidence of bony union is seen. Some surgeons routinely remove the plate and screws once bony healing has been achieved in anticipation of further surgery. There are limits to the extent of mechanical axis correction achievable with internally fixed osteotomies. Significant deformities or patients who require additional bone lengthening may be best managed with gradual correction using an external fixator according to Ilizarov's techniques of distraction osteogenesis [33,34].

The principal advantages of the HTO over alternative operations are the ability to correct deformity and the preservation of bone stock, which allows any future arthroplasty to be performed with relative simplicity. After osteotomy, patients are not usually requested to restrict their activities and the level of function is limited only by symptoms. Therefore an HTO is thought to be a good option for heavy and high-demand patients. Evidence suggests that the outcome of osteotomy is more predictable in males versus females [35].

As load is being transferred from one side of the knee to the other, HTO is relatively contraindicated in patients with OA affecting more than one compartment of the knee or pathology in the compartment into which load is transferred (for example, a significant meniscal tear). The osteotomy may increase forces at the patella-femoral joint and therefore is contraindicated in patients with significant patella-femoral symptoms or patella alta or baja. Before HTO patients must be counseled that they may notice a visible change in the alignment of their leg and a mild leg length discrepancy.

Isolated lateral compartment OA is seen less frequently. This may be managed with an osteotomy transferring the mechanical axis to the medial compartment according to the same biomechanical principles employed in an HTO for medial disease. Many patients with isolated lateral compartment OA have a pre-existing valgus (knocked knee) deformity that is typically secondary to an underdeveloped lateral femoral condyle. This means that correcting the deformity on the tibial side of the joint will lead to an oblique joint line, which is considered detrimental to the long-term function of the joint. For this reason the deformity is classically corrected by a distal femoral osteotomy (DFO). The literature suggests that the outcome of DFO is less predictable than that of HTO.

High tibial osteotomy should not be considered definitive treatment for an osteoarthritic knee but rather as a means of providing sufficient symptomatic relied to delay the need joint replacement. Survivorship, defined as time to arthroplasty, is reported variably in the literature but may be as high as 98% at 10 years and 70% at 20 years [36-38]. However, reviewing the literature as a whole the average survivorship is approximately 60% to 70% at 10 years.

A concern of HTO is its potential affect on future TKR. Some surgeons routinely remove metalwork from the knee in anticipation of future arthroplasty but there is little evidence to support this. Incisions must be carefully planned to avoid future wound healing problems and limitations to exposure. The literature suggests that the results of TKR after HTO are not significantly different from those of TKR in an unoperated knee [39]. Our experience is that while the results of TKR after HTO are good this is more technically demanding than a TKR in a 'virgin' knee.

### Unicompartment knee replacement

Gunston and Marmor introduced unicompartment knee replacements independently in the 1970s. Early designs consisted of a stainless steel runner and a track of high molecular weight polyethylene that was used in either a unicompartmental or bicompartmental joint replacement [40]. Early outcomes were disappointing, and consequently many surgeons chose to treat their patients with single compartment OA with a TKR or HTO. In 1988, Marmor published results demonstrating 21 out of 97 cases had failed at 10 to 13-year follow-up [41]. More recently there has been resurgence in UKR [42] that may have been driven by several publications showing much improved survival and satisfaction rates [43,44].

UKR has theoretical benefits over TKR, which include: preservation of bone stock and soft tissues, preservation of a more natural gait pattern and kinematics, improved range of motion, reduced operative time, and reduced incision size.

Schneider *et al. *[45] specifically assessed the outcome of UKR in the younger patient. A total of 28 patients under 60 years (average age 52 years) were followed up between 2 and 6 years. Pain relief and function were described as good or excellent in 90% of cases. Of note, activity levels as described by Tegner and Lysholm improved slightly from 2.3 points preoperatively to 2.7 points postoperatively.

There have been a few prospective studies randomizing patients to HTO or UKR and evaluating functional outcomes. Borjesson *et al. *[46] used the British Orthopaedic Association score, gait analysis, range of movement and patient satisfaction measures to compare patients aged 55 to 70 with moderate medial knee arthritis randomized to either HTO or UKR. No differences were noted between the groups other than at 3 months after surgery when there was a significant difference in the time-distance variable of gait in favor of UKR. This became insignificant at 1-year and 5-year follow-up.

Ivarsson and Gillquist [47] evaluated the rehabilitation programs of ten HTO and ten UKR patients with an average age of 63 years. Gait analysis was performed alongside Lysholm knee function scoring and measures of muscle strength. Assessment at 6 months revealed greater strength in the UKR group compared to the HTO group but this had equalized by 12 months. Lysholm function scores were greater in the UKR group but not with statistic significance. Gait analysis demonstrated increased maximal gait velocity and duration of single-leg support in the UKR group postoperatively versus preoperatively. No such difference was observed in the HTO cohort.

As with HTO, patients have to be carefully selected for UKR. The procedure is contraindicated in patients with an inflammatory arthropathy, the disease should be confined to one side of the joint, however, in some publishes series asymptomatic patellofemoral OA is not seen as a contraindication to unicompartmental tibio-femoral replacement. The knee must flex beyond 120° (to allow preparation of the femoral bone surface and insertion of the components), any varus or valgus deformity must be passively correctable and less than 15°, and there must be less than 5° of fixed flexion deformity. For some fixed-bearing tibial component designs, a weight limit of 114 kg has been set. Traditionally, an anterior cruciate ligament (ACL) deficient knee was considered a contraindication to UKR, but this has not been borne out in the literature for fixed bearing joint replacements [48].

The author's experience of revising UKR to TKR is that it is typically a straightforward procedure similar to a primary TKR. However, a failed UKR may be associated with significant bone loss making revision a more complicated undertaking. Specific revision implants may be required with metal or bone augmentation to areas of bone loss. The published literature suggests that revision of a failed UKR to a second UKR is associated with a threefold risk of further revision compared to patients revised to a TKR [49] and the authors would not advocate this treatment. Reports of the outcomes of UKR revised to TKR are variable but typically similar to those of revision TKR, which are inferior to primary TKR [50].

### Patellofemoral joint replacement

Osteoarthritis of the knee confined to the PFJ in isolation is seen in approximately 10% of patients, and most frequently in females. Along with UKR, PFJ replacements were introduced in the 1970s. Interest in their use has been renewed recently. We consider this option in patients with predominant symptoms of PFJ arthritis and radiographic evidence of OA isolated to the PFJ. This procedure has the same theoretical benefits as any UKR over TKR including reduced operative time, less invasive surgery, quicker postoperative rehabilitation and relative ease of revision. As the cruciate ligaments and femoro-tibial compartments are preserved knee kinematics and gait are better preserved [51]. The Bristol Knee Group [52] performed over 425 PFJ arthroplasties reporting 95.8% 5-year survivorship and improved function with Oxford knee scores increasing from 18 to 39 points out of 48. In 3% of cases maltracking was observed, some of which requiring distal alignment revision surgery. In all, 7% of cases developed disease in the medial or lateral tibiofemoral joint causing recurrent pain. A retrospective review comparing the outcomes of patients with an average age of 60 years undergoing PFJ replacement with patients receiving TKR found similar postoperative Knee Society Clinical Rating System scores but reduced blood loss and hospital stay in the PFJ group [53]. The authors concluded that isolate PFJ arthroplasty yields similar clinical outcomes to TKR but is a less invasive option for this select subgroup of patients. We believe that PFJ replacement is a viable option for patients with symptomatic isolated PFJ OA but we counsel patients that the long-term outcome of this procedure is as yet unclear and future revision to a TKR is likely.

## Minimally invasive techniques

### Arthroscopy

The role of arthroscopy in the treatment of OA is controversial. It is a safe, relatively straightforward treatment and therefore widely used. This use is not supported in the literature by clear evidence of a significant or lasting benefit. In a trial of 180 patients randomized to arthroscopic lavage, debridement, or placebo [54], no benefit was seen between surgery and placebo in terms of pain scores or function at 2-year follow-up. The average patient age was 53 years. Patients with OA may have associated symptomatic meniscal pathology but the contribution of this to overall levels of symptoms can be difficult to determine. The UK National Institute of Clinical Excellence (NICE) advise that arthroscopy, with or without debridement, should only be offered to patients as part of a treatment for OA if there is a clear history of 'locking, not gelling, or giving way', or radiographic evidence of loose bodies [55]. Arthroscopy for OA is not an entirely benign procedure and complications are described including reports of insufficiency fractures following knee arthroscopy. The pathological process appears to be subchondral fracture rather than osteonecrosis in this rare complication [56].

The authors believe that arthroscopy has a very limited role in the management of the osteoarthritic knee. We would consider arthroscopy only where there are clear signs and symptoms of meniscal pathology, a history consistent with true mechanical locking, or symptomatic loose bodies, superimposed on typical symptoms of OA.

### Microfracture

Microfracture is a minimally invasive technique that is performed arthroscopically. In patients with relatively small (1 to 2 cm^2^) and isolated chondral lesions the exposed subchondral bone is penetrated with an arthroscopic drill or awl with the aim of releasing pluripotent stem cells from the marrow. The hope is that the stem cells migrate into the resulting fibrin clot with the area being replaced with fibrous tissues, fibrocartilage, or hyaline-like cartilage. The technique has been described as 'quite effective' in treating non-weight bearing lesions in patients less than 35 years [57] but has poor outcomes in the osteoarthritic knee in the older patient [58]. The author's opinion is that this technique has little evidence to support its use in the management of significant OA.

### Chondrocyte implantation

Techniques of autologous chondrocyte implantation (ACI) and its variants, which include matrix-induced autologous chondrocyte implantation (MACI), are attracting great interest in the treatment of discrete chondral lesions. Such lesions usually are traumatic in origin but may with time lead to more widespread OA. The evidence for these techniques is mixed. A number of uncontrolled studies show symptomatic improvement following these techniques, however three well conducted randomized controlled trials (RCTs) compared ACI with microfracture and found no clinically significant difference beyond short-term follow-up [59-61]. All groups demonstrated 'hyaline-like' articular cartilage at biopsy. The authors believe that while these techniques may have a place in the management of isolated articular cartilage lesions that is yet to be firmly established, there is no evidence to support their use in more widespread OA of the knee.

## Summary

The management of the young patient with an osteoarthritic knee remains a significant challenge for the orthopedic surgeon. Multiple non-surgical treatments are available, but are unlikely to offer a lasting improvement in symptoms. We believe that with careful interrogation of patient's symptoms and a thorough examination it is usually possible to identify those osteoarthritic knees with coexisting pathology amenable to arthroscopic treatment, leading to an improvement in symptoms and delay of more invasive surgery.

Once these options are exhausted, the main choice is between HTO, UKR or TKR. While TKR remains an option that must be considered in the young patient the reduced longevity and higher expectations in this group allied to potential bone loss associated with a failed TKR mean that whenever possible we prefer to consider the options of HTO or UKR. Which option is most suitable depends upon patient characteristics and expectations.

In our practice, young, active and heavier males tend to be offered HTO. In young patients who fall outside this group our experience is that unicompartmental knee replacement is a successful procedure and we have had success revising these implants to TKR.

Autologous chondrocyte implantation and other techniques of 'biological' joint replacement are exciting treatment prospects for the future but are not currently supported by evidence.

## Abbreviations

ACI: autologous chondrocyte implantation; ACL: anterior cruciate ligament; DFO: distal femoral osteotomy; HA: hyaluronan; HTO: high tibial osteotomy; IA: intra-articular; MACI: matrix-induced autologous chondrocyte implantation; NSAID: non-steroidal anti-inflammatory drug; OA: osteoarthritis; PFJ: patello-femoral joint; TKR: total knee replacement; UKR: unicondylar knee replacement.

## Competing interests

The authors declare that they have no competing interests.

## Authors' contributions

Both EH and PS were responsible for drafting and revising the manuscript. Both authors have read and approved the manuscript for publication.

## Pre-publication history

The pre-publication history for this paper can be accessed here:

http://www.biomedcentral.com/1741-7015/11/14/prepub
